# Impact of the Pilot Volume-Based Drug Purchasing Policy in China: Interrupted Time-Series Analysis with Controls

**DOI:** 10.3389/fphar.2021.804237

**Published:** 2021-12-22

**Authors:** Yilin Chen, Xu Ji, Hong Xiao, Joseph M Unger, Yi Cai, Zongfu Mao, Kai Yeung

**Affiliations:** ^1^ The Comparative Health Outcomes, Policy, and Economics (CHOICE) Institute, University of Washington, Seattle, WA, United States; ^2^ Wuhan University Global Health Institute, Wuhan University, Wuhan, China; ^3^ Wuhan University School of Health Sciences, Wuhan, China; ^4^ Public Health Sciences Division, Fred Hutchison Cancer Research Center, Seattle, WA, United States; ^5^ SWOG Statistics and Data Management Center, Seattle, WA, United States; ^6^ Kaiser Permanente Washington Health Research Institute, Seattle, WA, United States

**Keywords:** volume-based purchasing, pharmaceuticals, price control, interrupted time series, impact evaluation, bulk purchasing

## Abstract

Centralizing procurement for prescription drugs has the potential to reduce drug spending by creating economies of scale and by improving purchasing power. In March 2019, the Chinese government launched a volume-based purchasing (VBP) pilot program using a competitive bidding process to purchase accredited generic drugs for which branded drug substitutes were available. We performed an interrupted time-series design to estimate the change in monthly drug purchase quantity and spending comparing 14 months before and 7 months after the VBP pilot. We obtained monthly prescription drug purchase data for all purchases from public medical institutions in the three large pilot cities (Beijing, Shanghai and Xi’an) and two non-pilot cities (Changsha and Zhengzhou) between January 2018 to September 2019. We used negative binomial regression and log-linked Gamma Generalized Linear Model for purchase quantity and spending respectively. We evaluated heterogeneity of impact by pilot city, drug type (selected or non-selected drugs), and therapeutic class (cardiovascular disease, mental disorder and cancer) separately. The implementation of the pilot reform was associated with a 132% (95%-CI: 104–165%, *p* < 0.001) increase in the purchase quantity of selected drugs in pilot cities compared to an 17% decrease (95%-CI: 9–25%, *p* < 0.001) in control cities. In contrast, the purchase quantity of branded and other drugs in pilot cities decreased by 38% (95%-CI: 27–46%, *p* < 0.001) and 77% (95%-CI: 71–81%, *p* < 0.001), respectively; while in control cities, these remained at similar levels. Overall, in pilot cities, there was a 35% (95%-CI: 28–41%, *p* < 0.001) decrease in the purchase spending for all drugs in the first post-policy month, from 8.1 billion CNY estimated in the absence of VBP down to 5.3 billion CNY; in control cities, the change was negligible. The largest reduction in spending occurred for drugs for the treatment of cardiovascular diseases. The evidence suggests a positive impact of the VBP pilot in reducing overall drug spending and increasing the use of accredited generics in three pilot cities. This overall trend is not observed in two non-pilot cities. Assessments of long-term impact of the VBP policy on additional key outcomes including drug prescriptions, drug utilization, patients’ health outcomes and payments on drugs are needed.

## Introduction

Growing healthcare spending remains a persistent challenge in many countries worldwide, especially in those experiencing rapid economic growth. In China, total drug spending has more than doubled from 754 billion CNY (120 billion USD) in 2009 to 1.82 trillion CNY (291 billion USD) in 2017. Pharmaceutical expenses in 2017 constituted 34% of total healthcare spending in China, much higher than most other Asian countries including Japan (18%) and South Korea (20%), and higher than the OECD average (16%) (China National Health Dev, 2018; (2021). Pharmaceutic, 2021). Thus, the Chinese Government is currently exploring strategies to contain rapidly growing healthcare spending (Chen and Schweitzer, 2008). Various price reduction strategies for pharmaceuticals have been considered globally, including pooled procurement, generic competition, international reference pricing, indication-based pricing, and outcomes-based contracting (Yeung et al., 2017; Verghese et al., 2019; Chen and Carlson, 2021; Mulcahy et al., 2021). Centralized procurement for medical products has yielded cost savings across many settings by creating economies of scale and improving purchasing power (Seidman and Atun, 2017). Volume-based purchasing (VBP) is expected to reduce pharmaceutical spending through three mechanisms: 1) pooled procurement process for drugs across multiple buyers increases negotiation power over pricing, and urges pharmaceutical companies to offer lower unit costs in exchange for a larger volume of purchase; 2) reduced operational expenses due to fewer negotiations and more bulk shipments; 3) potentially reshaped market share of pharmaceuticals from substituting branded with generic drugs and lower prices from generic drugs (World Health Organization, 2007; Waning et al., 2009; Lybecker, 2013).

A new VBP pilot (i.e., the “4 + 7” pilot) program in China was introduced in December 2018 and officially launched in March 2019. Under this VBP, the Chinese government centralizes the purchasing process nationwide and pre-defines the purchase quantity. Manufacturers compete on price by submitting bids. The VBP pilot was launched in 11 major cities based on large market shares (representing overall 1/3 of the national pharmaceutical market by volume) (State Council of China (2, 2019a), previous procurement reform experience, and willingness to participate. The VBP included 25 drugs (by generic name) that met pre-defined quality standards (Supplementary Table S1). Compared to pre-2018 practices, the current policy is characterized by: 1) organizing at the national level to increase purchasing power; 2) predefining procurement volumes with potential bidders as an incentive; and 3) Generic Quality Consistency Evaluation (GQCE) approval to ensure the quality of generic drugs (Huang et al., 2017a).

**FIGURE 1:**
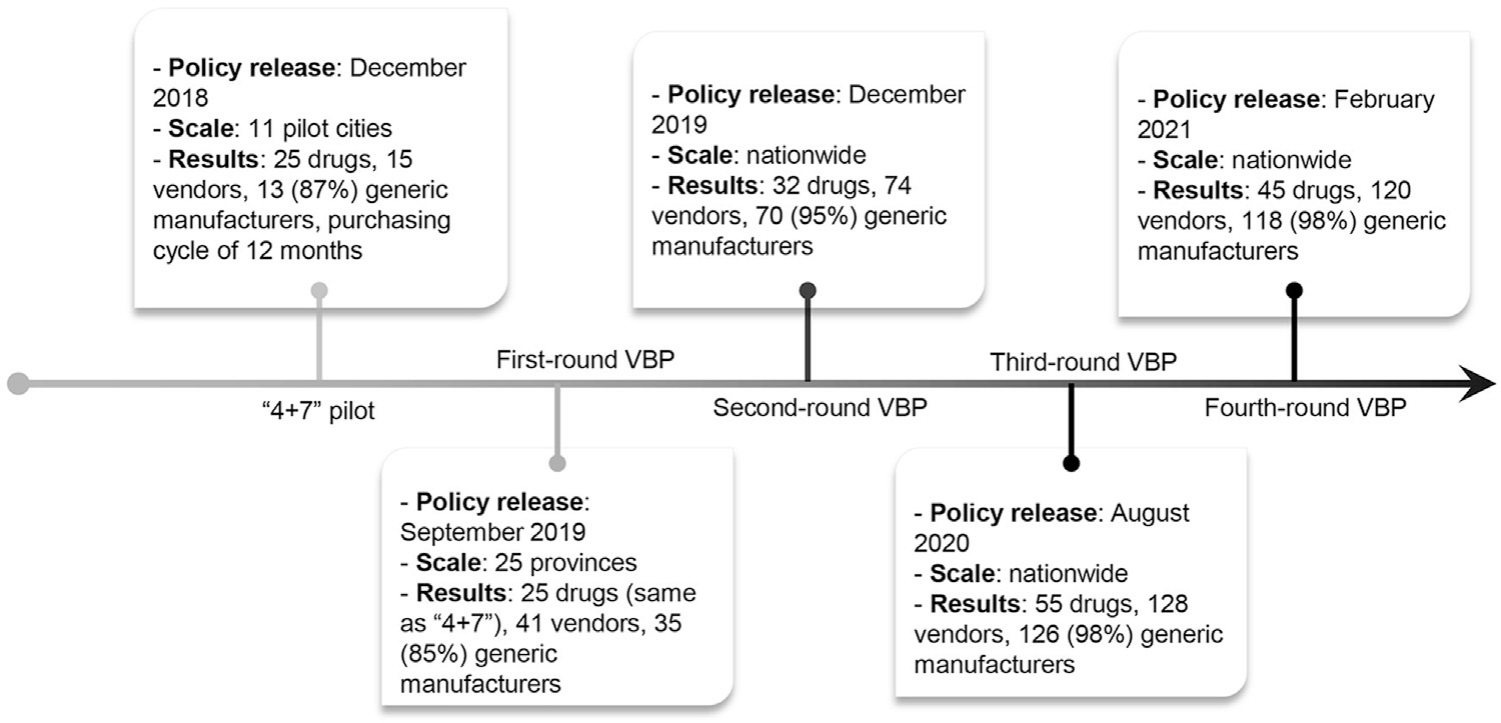
Timeline of the volume-based procurement reform. 11 pilot cities include 4 municipalities (i.e., Beijing, Tianjin, Shanghai, and Chongqing) and 7 cities (i.e., Shenyang, Dalian, Xiamen, Guangzhou, Shenzhen, Chengdu, and Xi’an).

On the basis of strong political commitments, several rounds of VBP have been successively implemented nationwide since the launch of the pilot program (Figure 1) (State Council of China (2, 2019b; National Medical Security, 2019), yet there is limited evidence regarding the impact of the VBP on drug spending and utilization. After the implementation of the pilot, the unit prices of 25 selected drugs in the 11 pilot cities dropped by 52% on average with the maximum unit price reduction of 96% (Tang et al., 2019a; Yue, 2019). Using a pre-post design and data from a single pilot city, Chen et al. found an overall 74% increase and 37% decrease in the quantity of and spending on the purchase of selected drugs (Chen et al., 2020). However, their analysis only compared the absolute change in the period before and after the policy implementation, without considering confounding by secular trends. In addition, most literature focused on the implementation process or policy effect in a single city. No studies have evaluated the implementation of the pilot in other large cities. Therefore, a more comprehensive and rigorous evaluation is crucial to evaluating the effectiveness of the pilot. Our study aims to evaluate the impact of the pilot on drug procurement using an interrupted time series analysis with controls in the three large pilot cities (i.e., Beijing, Shanghai, and Xi’an) with a total population of about 60 million. We also examine if the patterns of change associated with the reform differed by cities, drug types, and therapeutic categories. Since the majority of individuals seek care in public hospitals in China (Meng et al., 2019), and hospitals automatically convert prescriptions to selected generics, our analysis is generalizable to almost the entire population in these pilot cities.

## Materials and Methods

### Data

Data on the quantity and spending of drug procurement come from a database under the China Drug Supply Information Platform (CDSIP, http://cdsip.nhc.gov.cn/), covering all provincial drug centralized procurement platforms and purchasing records of public medical institutions (public hospitals and government-run community healthcare centers). The CDSIP is a national drug database constructed and maintained by the Statistical Information Center of the National Health Commission (NHC) of PRC, and was officially launched on October 22, 2015. Our investigators at Wuhan University Global Health Institute obtained data on monthly purchase of each of the 25 bid-winning drugs for pooled procurement (selected drugs) between January 2018 to September 2019. The three pilot cities included Beijing, Shanghai and Xi’an in mainland China. These cities were chosen to represent diverse geographical locations (located in north, east, and central China, respectively). Two comparable non-pilot cities (Changsha and Zhengzhou) were used to control for potential confounding factors that would affect both pilot and non-pilot cities. In addition to data on selected drugs, we also reviewed and extracted data on monthly purchase of corresponding substitute drugs, including originator (branded drugs), alternative drugs and other drugs. Alternative drugs are drug products with the same active pharmaceutical ingredients but produced by other non-winning pharmaceutical manufacturers. Other drugs are considered to be therapeutic equivalents with different active pharmaceutical ingredients but have the same clinical effect and safety profile when administered to patients under the conditions specified in the label. The source of these definitions is the Monitoring Plan Work of National Centralized Drug Procurement and Use issued by the NHSA of China (Wang and Wu, 2019). We obtained yearly estimates of population size from Statistical Yearbooks (National Bureau of Statis, 2021). The RECORD (REporting of studies Conducted using Observational Routinely-collected Data) guidelines were followed. This study was exempt from institutional ethical approval since results were presented at population level. The datasets generated and analyzed during the current study are not publicly available due to data use and non-disclosure agreement.

### Statistical Analysis

We examined changes in the pattern of drug procurement associated with the implementation of the pilot using interrupted time-series (ITS) design, a quasi-experimental design for strong causal inference in the evaluation of population-based health interventions introduced at a clear point in time (Wagner et al., 2002; Yeung et al., 2020; Xiao et al., 2021a). Two outcome variables were measured in this study: monthly purchase quantity and spending. Purchase quantity was measured by Defined Daily Doses (DDDs), which was developed by the WHO Collaborating Center for Drug Statistics Methodology and used in previous studies (World Health Organization, 2021a; World Health Organization, 2021b; Yang et al., 2021). Given a disperse variation structure in procurement quantity and skewness in spending, we used negative binomial regression and log-linked Gamma Generalized Linear Model (GLM) for purchase quantity and spending respectively. The GLM model equation was expressed as follows (Xiao et al., 2021a):

$$\log\left(E\left(Y_{t}\right)\right)=\beta_{0}+\beta_{1}T+\beta_{2}X_{t}+\beta_{3}\left(T-T_{0}\right)*X_{t}+\sum_{m=2}^{12}\beta_{m}M$$

where *Y*_*t*_ is the independent outcome variable (either monthly purchase quantity/spending) *T* and *T- T*_*0*_ is the time (month) since the start of the study (January 2018) and the time since the implementation of pilot program (*T*_*0*_: March 2019) respectively. *X*_*t*_ is an indicator variable where pre-intervention period is coded as 0 and post-intervention period is coded as 1. *M* is the indicator variable for calendar month used to account for seasonal variation. Each subgroup defined by intervention group (pilot cities versus non-pilot cities), intervention city (Beijing, Shanghai, Xi’an, Changsha and Zhengzhou), drug type (selected, originator, alternative and other drugs), and therapeutic category (cardiovascular disease, mental disorder and cancer) was analyzed separately. The three disease categories were chosen because they account for 17 out of the 25 pooled procurement drugs and are the leading causes of disability-adjusted life year in China (Zhou et al., 2019). To evaluate heterogeneous effects in subgroups, we examined how the main policy effect terms interacted with each subgroup (see the methods section in the Supplementary Appendix).

We decomposed the overall policy impact by reporting both the level change and slope change, which are represented by the coefficients *β*_*2*_ and *β*_*3*_, respectively (Wagner et al., 2002). Newey-West standard errors with autocorrelation of up to three lags were used within our models to accommodate serial autocorrelation in residual errors. Secondarily, the policy impact in the 7 months after implementation was calculated as the difference between estimated sum of monthly outcomes in the presence of the policy (i.e., factual estimate) and in the absence of the policy (i.e., counterfactual estimate) (Xiao et al., 2021b). To generate confidence intervals for the estimates of absolute and relative change, we simulated 10,000 predictions per month under each model (factual estimate and counterfactual estimate) using the coefficients and covariance matrix of each model using a multivariate normal distribution (Unger et al., 2021; Unger and Xiao, 2021). The 2.5 and 97.5 percentiles of the simulated values represented the 95% confidence interval. P-values were calculated as the smaller of the proportion of simulated values falling above or below zero (depending on the direction of the comparison), multiplied by two to present a 2-sided *p*-value.

Analyses were conducted in R-version-4.0.2 (R-Project for Statistical Computing) using data obtained on October 23, 2020. A 2-sided *p* <0 .05 indicated statistical significance.

## Results

**FIGURE 2:**
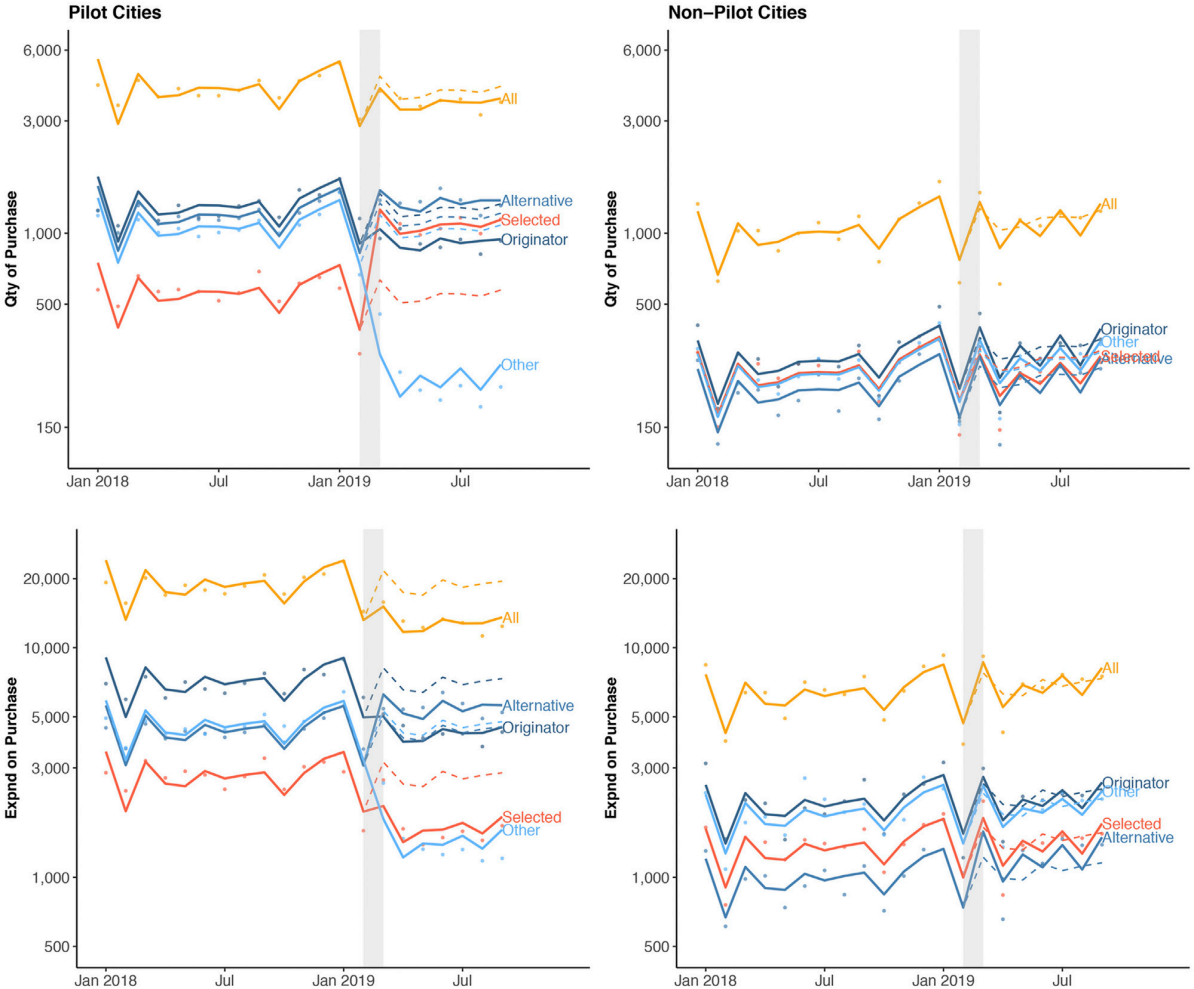
Quantity of and spending on drug purchase stratified by cities and drug categories over time. Dots indicate observed monthly purchase quantity (Qty, measured in DDD) or purchase spending (Expnd, measured in CNY) per 1, 000 population. The solid lines show the model fitted regression line, and the dashed lines represent model-estimated expected (i.e., counterfactual) purchase had the pilot program not occurred. The observed nadir in drug purchases in a calendar year coincides with the annual lunar new year in February.

In total, 20.5 billion CNY was spent on 481.5 billion drugs in pilot cities, and 2.6 billion CNY was spent on 41.3 million drugs in non-pilot cities from January 2018 to September 2019 (Supplementary Table S2). We observed similar pre-policy monthly trends in purchase quantity and spending in the intervention and control groups. An overall increasing trend in the purchase of all drug categories (selected, originator, alternative and other drugs) was observed in the pre-pilot phase in both pilot and non-pilot cities (Figure 2, Supplementary Figure S1,S2). Observed monthly purchase quantity and spending for the study drugs in the pilot cities dropped by 2.8 million (11.7%) and 331 million CNY (30.4%) post-policy, respectively. In contrast, monthly purchase quantity and spending in the non-pilot cities did not change post-policy (Supplementary Table S2).

**FIGURE 3:**
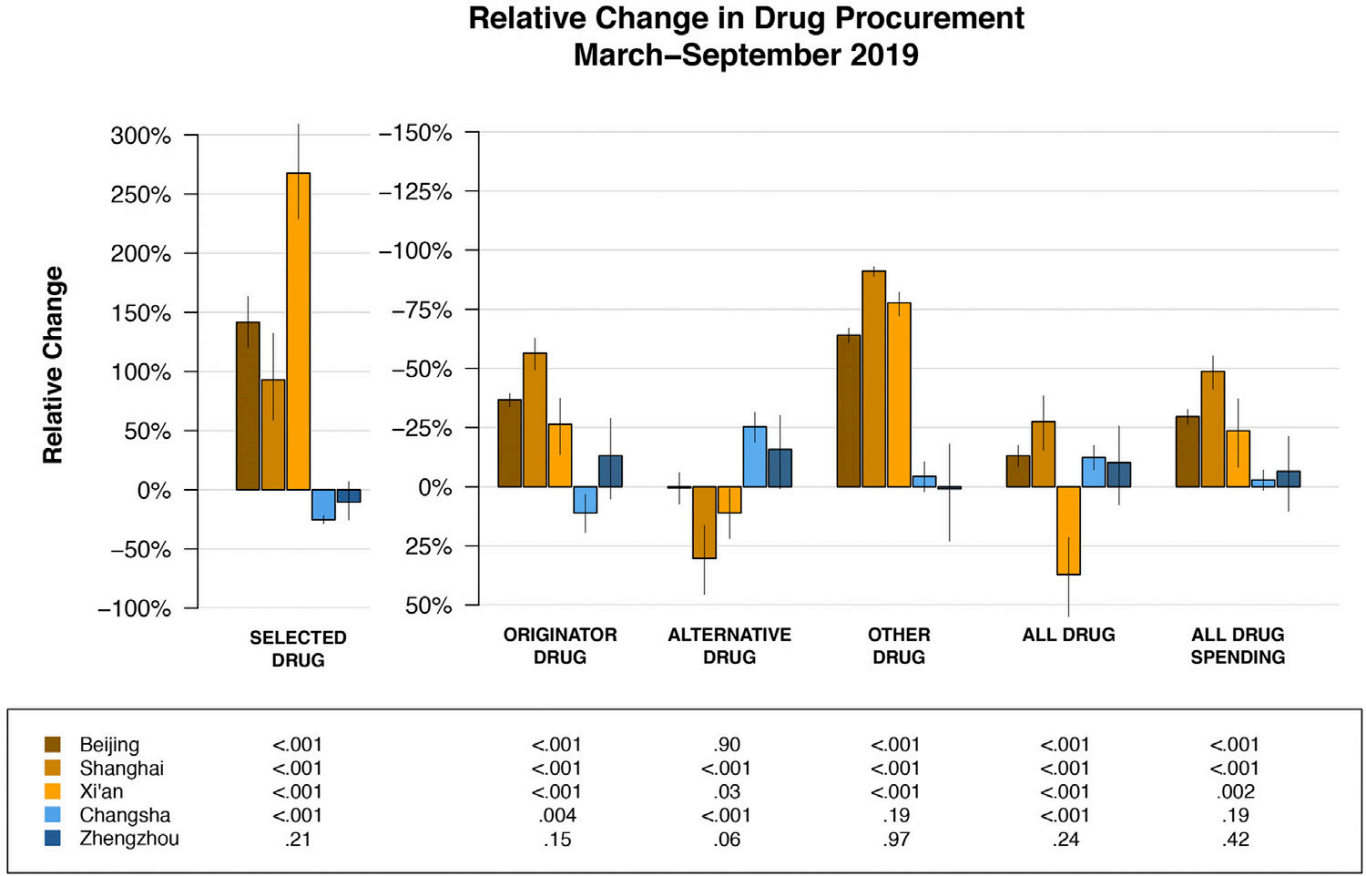
Relative change in drug purchase compared to expected purchase (had the pilot reform not occurred) by cities and drug categories. Specific point estimates for relative change and the corresponding 95% confidence intervals are provided in Supplementary Table S4.

### Level and Slope Changes in Drug Quantity and Spending

In the first post-policy month (representing the model-estimated level change), there was an increase in purchase quantity for the selected drugs in pilot cities (relative risk (RR) = 2.32, 95%-CI: 2.04–2.65, *p* < 0.001) (Table 1). In contrast, the purchase quantity decreased by 38% (RR = 0.62, 95%-CI: 0.54–0.73, *p* < 0.001) for originator drugs and 77% (RR = 0.23, 95%-CI: 0.19–0.29, *p* < 0.001) for other drugs, respectively. Combined with the 13% increase in the purchase quantity of alternative drug (RR = 1.13, 95%-CI: 1.03–1.23, *p* < 0.001), this led to a 15% (RR = 0.85, 95%-CI: 0.78–0.94, *p* < 0.001) decrease in the purchase quantity and 35% (RR = 0.65, 95%-CI: 0.59–0.72, *p* < 0.001) decrease in the purchase spending for all drugs in the first post-policy month. In contrast, the purchase quantity and spending for all drugs remain similar post-policy in the non-pilot cities.

**TABLE 1:** Interrupted time-series regression model estimates.

	Immediate change		Monthly change	
	RR (95% CI)	*p*-value	RR (95% CI)	*p*-value
Pilot Cities				
Selected Drugs^a^	2.32 (2.04, 2.65)	<0.001	1.00 (0.99, 1.00)	0.978
Originator^a^	0.62 (0.54, 0.73)	<0.001	1.00 (0.99, 1.02)	0.432
Alternative Drug^a^	1.13 (1.03, 1.23)	0.011	1.01 (1.00, 1.01)	0.054
Other Drug^a^	0.23 (0.19, 0.29)	<0.001	0.99 (0.98, 1.00)	0.020
All Non-selected Drug^a^	0.67 (0.61, 0.75)	<0.001	1.00 (1.00, 1.01)	0.319
All Drugs^a^	0.85 (0.78, 0.94)	<0.001	1.00 (1.00, 1.01)	0.444
All Drug (Spending)	0.65 (0.59, 0.72)	<0.001	1.00 (1.00, 1.01)	0.571
Non-pilot Cities				
Selected Drugs^a^	0.83 (0.75, 0,91)	<0.001	0.99 (0.96, 1.02)	0.508
Originator^a^	0.97 (0.82, 1.14)	0.681	0.99 (0.95, 1.03)	0.518
Alternative Drug^a^	0.79 (0.66, 0.94)	0.008	0.99 (0.95, 1.03)	0.636
Other Drug^a^	1.04 (0.87, 1.23)	0.691	0.99 (0.96, 1.02)	0.497
All Non-selected Drug^a^	0.87 (0.71, 1.06)	0.172	0.99 (0.95, 1.03)	0.539
All Drugs^a^	0.83 (0.63, 1.08)	0.156	0.99 (0.95, 1.03)	0.528
All Drug (Spending)	0.86 (0.70, 1.06)	0.157	0.99 (0.95, 1.02)	0.415

a Monthly quantity of purchase.

Note: Pilot cities include Beijing, Shanghai and Xi’an, and Non-pilot cities include Changsha and Zhengzhou unless specified otherwise.

Immediate change refers to the change in March 2019, Monthly change refers to the gradual change from April-September 2019.

Non-selected drugs include originator, alternative, and other drugs.

Patterns of change in the purchase quantity of selected, originator, and other drugs were similar among the three pilot cities (Supplementary Figure S1). The immediate increase in the purchase quantity of selected drugs was evident in all three pilot cities: Beijing (RR = 2.55, 95%-CI: 2.29–2.82, *p* < 0.001), Shanghai (RR = 2.08, 95%-CI: 1.69–2.56, *p* < 0.001) and Xi’an (RR = 2.26, 95%-CI: 1.86–2.74, *p* < 0.001), respectively. In contrast, the immediate decrease in originator and other drugs were substantial in all three cities (Supplementary Table S3). Over the seven post-policy months (representing the slope change), we only observed significant monthly increase in Xi’an (RR = 1.17, 95%-CI: 1.13–1.22, *p* < 0.001) in the purchase quantity of selected drugs. The gradual monthly change in the purchase quantity of selected drugs was negligible in Beijing (RR = 0.98, 95%-CI: 0.97–0.99, *p* = 0.006) and Shanghai (RR = 0.97, 95%-CI: 0.92–1.03, *p* = 0.372). All three cities have decreased purchase spending for all drugs in the first post-policy month.

### Overall Policy Effects in Drug Quantity and Spending

During the 7-month post-policy period, the estimated number of selected drugs purchased in pilot cities was 44.6 million. In contrast, the estimated number of selected drugs purchased in pilot cities had the reform not occurred was 19.2 million (Table 2, Supplementary Figure S3). Therefore, the policy was associated with an increase of 25.4 million (232.9% of expected, 95%-CI: 207.1–260.9%, *p* < 0.001). Over that same period, the estimated spending on all drugs in pilot cities was 5.3 billion CNY compared to 8.1 billion CNY had the reform not occurred (65.4% of expected, 95%-CI: 59.0–72.2%, *p* < 0.001). In contrast, the change in the purchase quantity and spending for any drug type in non-pilot cities was negligible.

**TABLE 2:** Overall changes in purchase stratified by drug and intervention groups, March- September 2019.

City	Drug	Expected	Model-fitted actual	Absolute change			Relative change (%)			*p*-value
				Estimate	95% CI		Estimate	95% CI		
**Piot cities**										
	Selected	1,920	4,457	2,536	2,300	2,759	132.9	107.1	160.9	<0.001
	Originator	6,205	3,748	−2,457	−2,941	-1990	−39.5	−44.4	−34.3	<0.001
	Alternative	5,058	5,527	470	74	857	9.4	1.4	18.0	0.021
	Other	4,154	1,004	−3,150	−4,005	-2,424	−75.6	−80.6	−69.8	<0.001
	Non-selected	15,324	10,291	−5,033	−6,531	-3,607	−32.7	−39.1	−25.7	<0.001
	All	17,278	14,747	−2,531	−4,242	−949	−14.5	−22.5	−6.0	0.002
	All (spending)	80,827	52,753	−28,074	−35,940	−20,668	−34.6	−41.0	−27.8	<0.001
**Non-pilot Cities**										
	Selected	372	339	−33	−59	−8	−8.8	−14.9	−2.2	0.010
	Originator	397	430	33	−3	69	8.6	−0.7	18.8	0.072
	Alternative	365	326	−39	−66	−14	−10.7	−17.3	−3.9	0.005
	Other	358	390	32	−2	66	9.2	−0.6	19.9	0.067
	Non-selected	1,118	1,146	27	−68	120	2.6	−5.7	11.4	0.056
	All	1,496	1,484	−12	−133	105	−0.7	−8.3	7.4	0.861
	All (spending)	8,777	9,282	506	−27	1,020	5.8	−0.3	12.0	0.062

Note: Quantity of purchase is presented in 10, 000 DDD, expenditure on purchase is presented in 100, 000 CNY. Non-selected drugs include originator, alternative, and other drugs.

Heterogeneity of overall policy effect across pilot cities were observed. The relative increase in the purchase quantity of selected drugs were statistically significantly greater in Beijing (242.5% of expected, 95%-CI: 220.3–263.5%, *p* < 0.001) and Xi’an (367.5% of expected, 95%-CI: 328.8–409%, *p* < 0.001) than those in Shanghai (92.9% of expected, 95%-CI: 158.7–232.2%, *p* < 0.001); whereas the decrease in the purchase spending on all drugs in Shanghai (51.3% of expected, 95%-CI: 44.7–58.6%, *p* < 0.001) was the greatest among the three pilot cities (Figure 3, Supplementary Table S4).

### Changes in Drug Quantity and Spending by Disease Category

The magnitude of the immediate increase in the purchase quantity of selected drugs was similar for cardiovascular diseases (RR = 2.53, 95%-CI: 2.62–2.82, *p* < 0.001) and cancer (RR = 2.18, 95%-CI: 1.56–3.05, *p* < 0.001) in pilot cities (Table 3). There was a smaller immediate increase (RR = 1.19, 95%-CI: 1.01–1.41, *p* = 0.035) in the purchase quantity of selected drugs for the treatment of mental disorders. For the originator and other drugs for cardiovascular diseases in pilot cities, there was a 35% (RR = 0.65, 95%-CI: 0.60–0.70, *p* < 0.001) and 72% (RR = 0.28, 95%-CI: 0.22–0.35, *p* < 0.001) decrease, respectively, in the purchase quantity in first post-policy month. There was an 26% (RR = 1.26, 95%-CI: 1.20–1.33, *p* < 0.001) increase in the purchase quantity of originator drugs for cancer. In pilot cities, the purchase quantity of all drugs for cardiovascular diseases in first post-policy month decreased by 10% (RR = 0.90, 95%-CI: 0.82–0.97, *p* = 0.010), with the spending decreased by 31% (RR = 0.69, 95%-CI: 0.64–0.75, *p* < 0.001). In contrast, the immediate change in the purchase spending for all drugs targeting cancer and mental disorder in March was not statistically significant. In non-pilot cities, the immediate or gradual change in the purchase of any drug category for any of the three disease types was either not statistically significant or only marginally statistically significant.

**TABLE 3:** Interrupted time-series regression model estimates (stratified by drug and disease categories).

	Pilot cities^a^				Non-pilot cities^a^			
	Immediate change		Monthly change		Immediate change		Monthly change	
	RR (95% CI)	*p*-value	RR (95% CI)	*p*-value	RR (95% CI)	*p*-value	RR (95% CI)	*p*-value
Selected Drug^b^	—	—	—	—	—	—	—	—
Cardiovascular disease	2.53 (2.62, 2.82)	<0.001	1.00 (0.98, 1.02)	0.928	1.06 (0.85, 1.31)	0.617	0.96 (0.90, 1.03)	0.287
Mental disorder	1.19 (1.01, 1.41)	0.035	0.96 (0.94, 0.99)	0.002	0.84 (0.67, 1.06)	0.143	0.88 (0.82, 0.94)	<0.001
Cancer	2.18 (1.56, 3.05)	<0.001	1.02 (0.93, 1.10)	0.707	1.78 (0.84, 3.79)	0.135	0.96 (0.86, 1.07)	0.432
Originator^b^	—	—	—	—	—	—	—	—
Cardiovascular disease	0.65 (0.60, 0.70)	<0.001	0.98 (0.96, 0.99)	<0.001	1.32 (1.02, 1.70)	0.035	0.88 (0.81, 0.96)	0.006
Mental disorder	1.06 (0.92, 1.21)	0.416	0.93 (0.91, 0.95)	<0.001	1.12 (0.80, 1.58)	0.504	0.99 (0.93, 1.05)	0.678
Cancer	1.26 (1.20, 1.33)	<0.001	0.99 (0.98, 1.00)	0.022	0.95 (0.68, 1.32)	0.759	1.11 (1.02, 1.20)	0.011
Alternative Drug^b^	—	—	—	—	—	—	—	—
Cardiovascular disease	1.10 (1.02, 1.19)	0.130	0.99 (0.97, 1.00)	0.128	0.95 (0.78, 1.15)	0.585	0.93 (0.88, 0.99)	0.019
Mental disorder	1.43 (1.17, 1.74)	<0.001	0.97 (0.92, 1.03)	0.387	1.03 (0.80, 1.32)	0.827	0.95 (0.88, 1.02)	0.162
Cancer	1.00 (0.94, 1.07)	0.988	0.98 (0.97, 0.99)	<0.001	1.11 (0.87, 1.43)	0.408	1.05 (1.00, 1.09)	0.030
Other Drug^b^	—	—	—	—	—	—	—	—
Cardiovascular disease	0.28 (0.22, 0.35)	<0.001	0.88 (0.85, 0.92)	<0.001	1.22 (1.01, 1.49)	0.043	0.94 (0.89, 1.00)	0.035
Mental disorder	0.64 (0.59, 0.69)	<0.001	0.94 (0.93, 0.96)	<0.001	1.22 (1.03, 1.46)	0.025	0.92 (0.87, 0.97)	0.002
Cancer	0.90 (0.82, 0.98)	0.021	0.96 (0.95, 0.97)	<0.001	—	—	—	—
All Non-selected drug^b^	—	—	—	—	—	—	—	—
Cardiovascular disease	0.72 (0.66, 0.78)	<0.001	0.97 (0.97, 0.98)	<0.001	1.16 (0.94, 1.44)	0.169	0.91 (0.85, 0.98)	0.012
Mental disorder	1.09 (0.93, 1.27)	0.297	0.96 (0.92, 1.01)	0.139	1.11 (0.90, 1.37)	0.320	0.94 (0.88, 1.00)	0.047
Cancer	0.96 (0.89, 1.03)	0.236	0.97 (0.96, 0.98)	<0.001	—	—	—	—
All Drug^b^	—	—	—	—	—	—	—	—
Cardiovascular disease	0.90 (0.82, 0.97)	0.010	0.98 (0.97, 0.99)	<0.001	1.14 (0.92, 1.41)	0.237	0.92 (0.86, 0.99)	0.025
Mental disorder	1.13 (1.02, 1.25)	0.018	0.96 (0.94, 0.99)	0.013	1.10 (0.81, 1.23)	0.999	0.91 (0.86, 0.98)	0.008
Cancer	1.04 (0.95, 1.13)	0.398	0.98 (0.97, 1.00)	0.018	—	—	—	—
All drug (Spending)	—	—	—	—	—	—	—	—
Cardiovascular disease	0.69 (0.64, 0.75)	<0.001	0.96 (0.96, 0.97)	<0.001	1.15 (0.93, 1.42)	0.203	0.92 (0.86, 0.99)	0.020
Mental disorder	0.98 (0.91, 1.06)	0.641	0.96 (0.93, 0.98)	<0.001	1.17 (0.99, 1.38)	0.061	0.91 (0.87, 0.96)	<0.001
Cancer	1.06 (1.00, 1.12)	0.056	1.02 (1.00, 1.03)	0.023	—	—	—	—

a Pilot cities include Beijing, Shanghai and Xi’an, and non-pilot cities include Changsha and Zhengzhou unless specified otherwise.

b Monthly quantity of purchase.

Note: For cancer treatment drugs, pilot city includes Beijing, non-pilot cities include Changsha (selected drug) and Zhengzhou (originator and other drug). Immediate change refers to the change in March 2019, Monthly change refers to the gradual change from April-September 2019. Non-selected drugs include originator, alternative, and other drugs.

## Discussion

Over the first 7 months of implementation, we found that the VBP pilot program resulted in an increase in the purchase of accredited generics in place of lower quality generics and off-patent branded drugs, resulting in lower total drug purchasing costs. The VBP pilot was designed to achieve lower unit prices through competitive bidding between accredited generic drug manufacturers, while also increasing market share of the quantity of these drugs at the same time, thereby driving down spending of all drugs. We observed an increase of 25.4 million (232.9% of expected) in purchased quantity of selected drugs and a decrease of 2.8 billion CNY (65.4% of expected) in spending on all drugs in three pilot cities, suggesting weaker policy effect in quantity and more profound effect in spending than estimates reported by Chen et al. in Shenzhen city (1,638.2% increase in purchased quantity of selected drugs and 37% decrease in spending on all policy-related drugs) (Chen et al., 2020). The difference in magnitude of change may be due to their different method of estimating the year-over-year growth rate from 2018 to 2019. Our findings are consistent with the impact of bulk purchasing policies among public payers (World Health Organization, 2007; Waning et al., 2009; Lybecker, 2013). New Zealand controlled pharmaceutical expenditures in the early 20th century through a combination of strong negotiation, bundling agreements, tendering sole-supply, and contracts. It resulted in immediate savings on pharmaceutical expenditures with up to 90 percent on some drugs, despite a 50% increase in volumes (Lybecker, 2013).

We observed regional differences of the policy effects on the purchase quantity and spending in the three pilot cities. The relative increase in the purchase quantity of selected drugs ranged from 92.9 to 367.5% of expected, with Xi’an having the greatest increase. While the relative decrease in purchase spending on all drugs ranged from 51.3 to 70.4% of expected, with the biggest decrease in Shanghai mainly due to its greater decrease in more expensive branded drugs than that in Xi’an and Beijing. Over the seven post-policy months, significant monthly increase in the purchase quantity of selected drugs was only observed in Xi’an. It is not unusual that the different local governments of the pilot cities adopted and implemented policies in different speeds and/or scopes. It’s likely that the baseline drug use structure in public hospitals varies by cities. For cities which have recorded higher use of innovative drugs historically, observed decrease in drug spending is likely to be more significant (Huang et al., 2017b; Tang et al., 2019b). In addition, insufficient payment capacity of local health insurance funds has been a challenge to some cities (Liu, 2018). It has been reported in closed-door briefings that complaints have been received because some provinces had pressure to pre-pay 30% of the drug purchasing fee due to deficit in the health insurance fund. If drug lists expand in further rounds of VBP, pressure may continue to mount (Sunshine Medical Procurem, 2019).

Policy effects of the pilot varied by disease categories. Drugs for the treatment of cardiovascular diseases made up the biggest category in the drug list, and the pilot has achieved greater increase in the purchase quantity of selected drugs in place of originators and other drugs, resulting in the decreased spending of all drugs for the treatment of cardiovascular diseases. Patents of many branded cardiovascular agents expired a long time ago, in the face of an emerging competitive generic market in China. The smaller policy effects of the pilot observed in mental disorder and cancer markets may be due to the fewer eligible drugs in these two disease areas that made to the negotiations. Mental health drugs tend to have less market competition because of fewer generic drug manufacturers, as well as less willingness for healthcare providers to switch patients. The innovative cancer drugs in the Chinese market remained strong, with a 26% increase in the purchase quantity in first post-policy month.

While promising, policy makers should consider additional factors in future iterations of VBP. This VBP was intended for the universe of generic drugs that only differ from branded drugs in cost and not on any clinical concerns. Policy makers should consider additional factors as well, such as the burden of diseases, patients’ need, or cost-effectiveness evidence. As China moves towards value-based pricing, cost and quality evidence can be used to inform the selection of drugs for pooled procurement (Li et al., 2018). Another possible consequence of the frequent renegotiations associated with the VBP could be abrupt changes in the preferred drug list, which could lead to lower patient satisfaction and potential adverse health outcomes. This would be especially the case if different clinical effectiveness and safety profiles are present, such as reduced drug adherence resulting in expensive subsequent treatments and hospital admissions (Mao et al., 2018). Drug shortages could happen if relying on a single source for supply, especially if more companies exit the market who would have been available to provide immediate back-up production (Zhang, 2019). For example, supply shortage has been reported since the second around of pooled procurement (Sunshine Medical Procurem, 2020). Later rounds of VBP may continue to improve the bidding rules to prevent future shortages due to sudden changes in the list of drugs and suppliers, especially during the COVID-19 pandemic.

There are also longer-term quality concerns. Lowering drug prices may cause some drug suppliers to reduce quality standards; further, those losing a bid may stop manufacturing and exit the market (Hu et al., 2015). The initial governmental proposal (which was set without any formal calculation) was for a price cut of 10% off previous lowest price for a given active ingredient. Strikingly, researchers have found the average unit price drop of 52% during negotiation compared with the minimum purchase unit price in 2017 (Tang et al., 2019a; Yue, 2019). The National Healthcare Security Administration (NHSA), as the biggest buyer, has a strong power to negotiate drug prices. However, the NMPA is responsible for quality assurance and the NHC takes charge of the rational use of medicines. In addition, the NHSA and the NHC monitor different levels of medication use. The former focuses on improving the secure and efficient use of health insurance funds in paying for medicines; while the latter is in charge of improving the rational use of medicines among providers (Liu et al., 2017). The current management systems need to be strengthened to improve its coordination mechanism and regulate drug quality and safety at various levels.

There are several limitations to this study. First, because the dataset spans a relatively short post-intervention period, our findings only reflected the impact of the VBP policy in the pilot phase and may not generalize to further reform and all settings. More rounds of VBP have been rapidly carried out in the country. Although early-stage evaluation could guide policy makers, healthcare providers and patients to better understand the reform and adapt accordingly, assessing further rounds of large-scale VBP programs after the pilot is equally important. Additionally, the 14-month pre-pilot data may not be sufficient to model the pre-pilot or counterfactual post-pilot trends. However, the consistently observed trends and seasonal patterns in the purchase of drugs of any category in all pilot and non-pilot cities, and, to our knowledge, the lack of uncontrolled potential time-varying confounders, suggest that our assumption about the counterfactual post-pilot based on existing information is valid. Lastly, our analysis only focused on drug purchase quantity and health facilities’ drug spending. Further research assessing the impact of the VBP policy on other outcomes, including drug prescription/utilization patterns, quality of drug, patients’ health outcomes and payments on drugs, as well as the landscape of pharmaceutical industry in China is needed.

China’s new VBP policy has demonstrated initial success in containing drug prices and reshaping the composition of the Chinese pharmaceutical market in favor of generics of high quality and low prices. Future studies are needed to assess further rounds of large-scale VBP programs, and the long-term impact on additional key outcomes including drug utilization, patient outcomes, and pharmaceutical research and development.

## Data Availability

The original contributions presented in the study are included in the article/Supplementary Material, further inquiries can be directed to the corresponding authors.
