# Supplementary figures and images for: Impact of the Pilot Volume-Based Drug Purchasing Policy in China: Interrupted Time-Series Analysis with Controls
Source: Front Pharmacol. 2021 Dec 22;12:804237. doi: 10.3389/fphar.2021.804237 (PMC9262040; doi:10.3389/fphar.2021.804237)

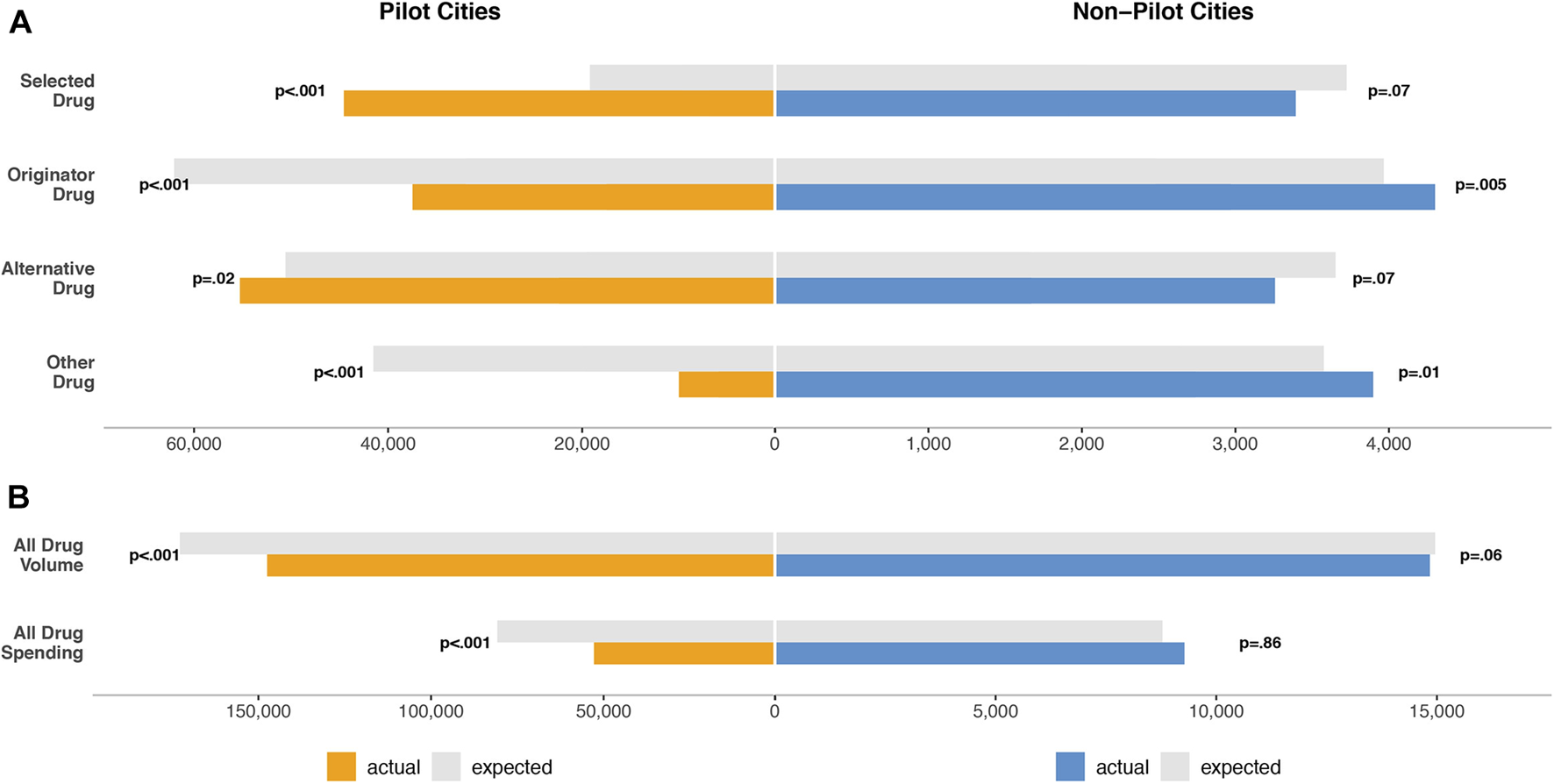

Supplement: Supplementary file 1 [file Image3.tif]

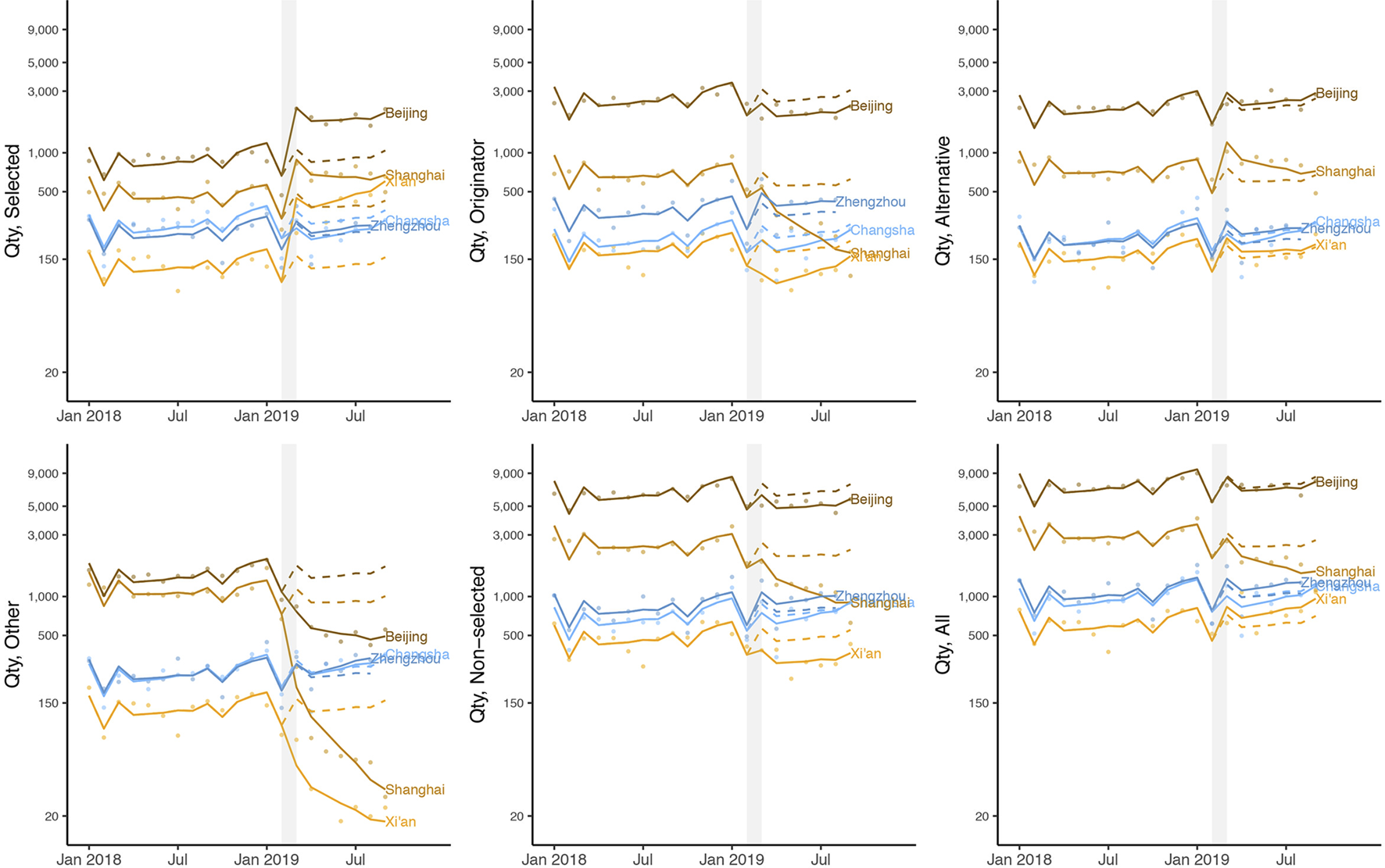

Supplement: Supplementary file 2 [file Image1.tif]

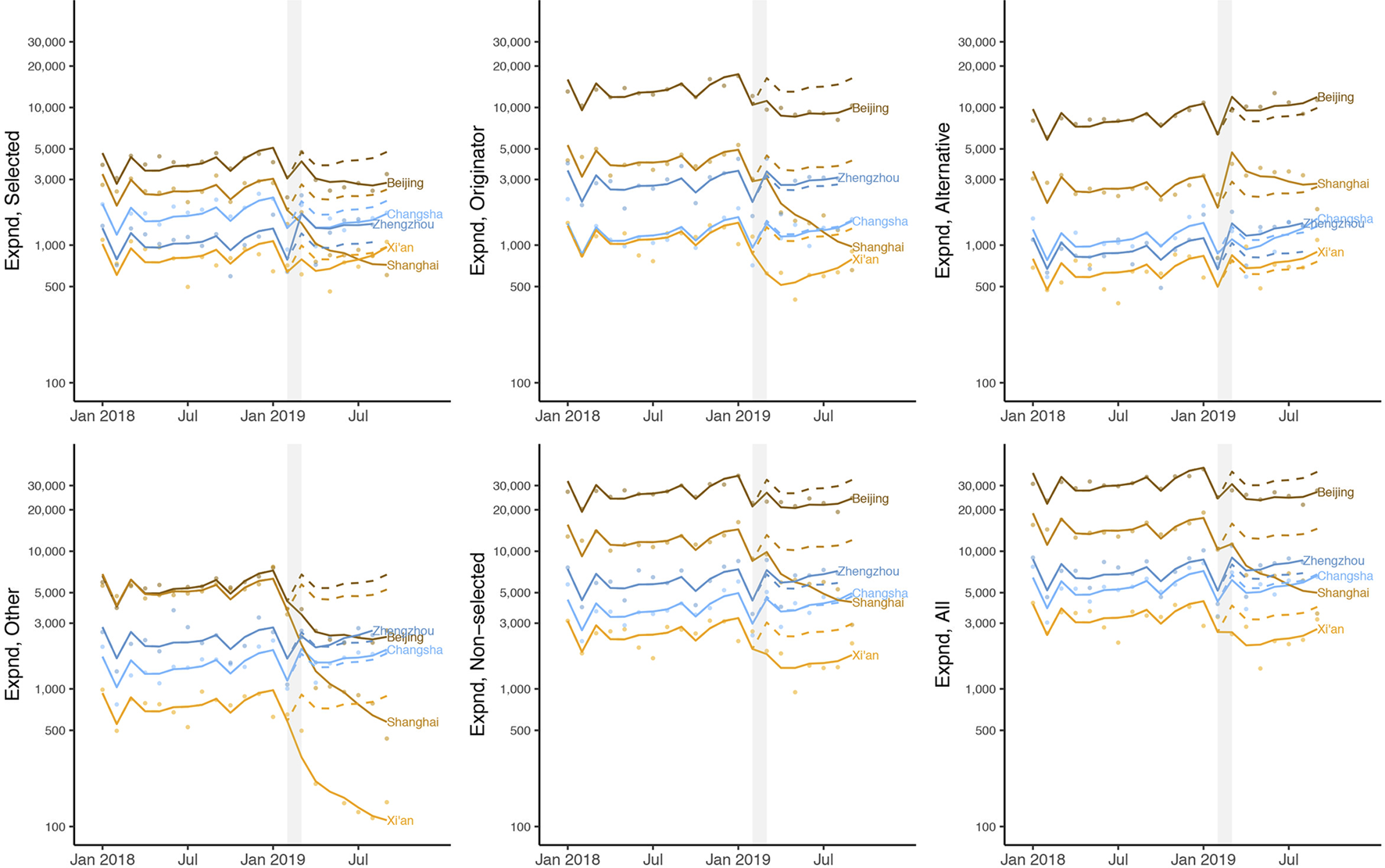

Supplement: Supplementary file 5 [file Image2.tif]
