# Supplementary material for: Impact of the Pilot Volume-Based Drug Purchasing Policy in China: Interrupted Time-Series Analysis with Controls
Source: Front Pharmacol. 2021 Dec 22;12:804237. doi: 10.3389/fphar.2021.804237 (PMC9262040; doi:10.3389/fphar.2021.804237)
Supplement: Supplementary file 4 [file DataSheet1.pdf]

Supplementary Online Content

Table S1. List of 25 VBP pilot drugs

Table S2. Observed average monthly drug purchase before and after the pilot program

Table S3. Interrupted time-series regression model estimates (stratified by drug categories and cities)

Table S4. Overall changes in purchase stratified by drug and cities, March- September 2019

Figure S1. Quantity of drug purchase stratified by cities and drug categories over time.

Figure S2. Spending on drug purchase stratified by cities and drug categories over time.

Figure S3. Expected (counterfactual estimate) versus actual (factual estimate) purchase by drug categories, March-September 2019.

Methodology: Statistical model evaluating heterogeneous effects in subgroups

The RECORD Statement

This supplementary material has been provided by the authors to give readers additional information about their work.

Table S1. List of 25 VBP pilot drugs

No	Drug name	Disease	Patent expiration year (US)
1	Flurbiprofen axetil	analgesics	1991
2	Dexmedetomidine	analgesics	2014
3	Cefuroxime axetil	antibiotics	2013
4	Montelukast	asthma	2012
5	Gefitinib	cancer	2017
6	Imatinib	cancer	2016
7	Pemetrexed	cancer	2017
8	Atorvastatin	cardiovascular disease	2011
9	Rosuvastatin	cardiovascular disease	2013
10	Clopidogrel	cardiovascular disease	2012
11	Irbesartan	cardiovascular disease	2011
12	Amlodipine	cardiovascular disease	2007
13	Fosinopril	cardiovascular disease	2003
14	Irbesartan and hydrochlorothiazide	cardiovascular disease	2011
15	Lisinopril	cardiovascular disease	2002
16	Losartan	cardiovascular disease	2009
17	Enalapril	cardiovascular disease	2000
18	Smectite	diarrhea	-
19	Entecavir	hepatitis B	2015
20	Tenofovir disoproxil	hepatitis B	2017
21	Escitalopram	mental disorders	2011
22	Paroxetine	mental disorders	2001
23	Olanzapine	mental disorders	2013
24	Risperidone	mental disorders	2007
25	Levetiracetam	seizure	2011

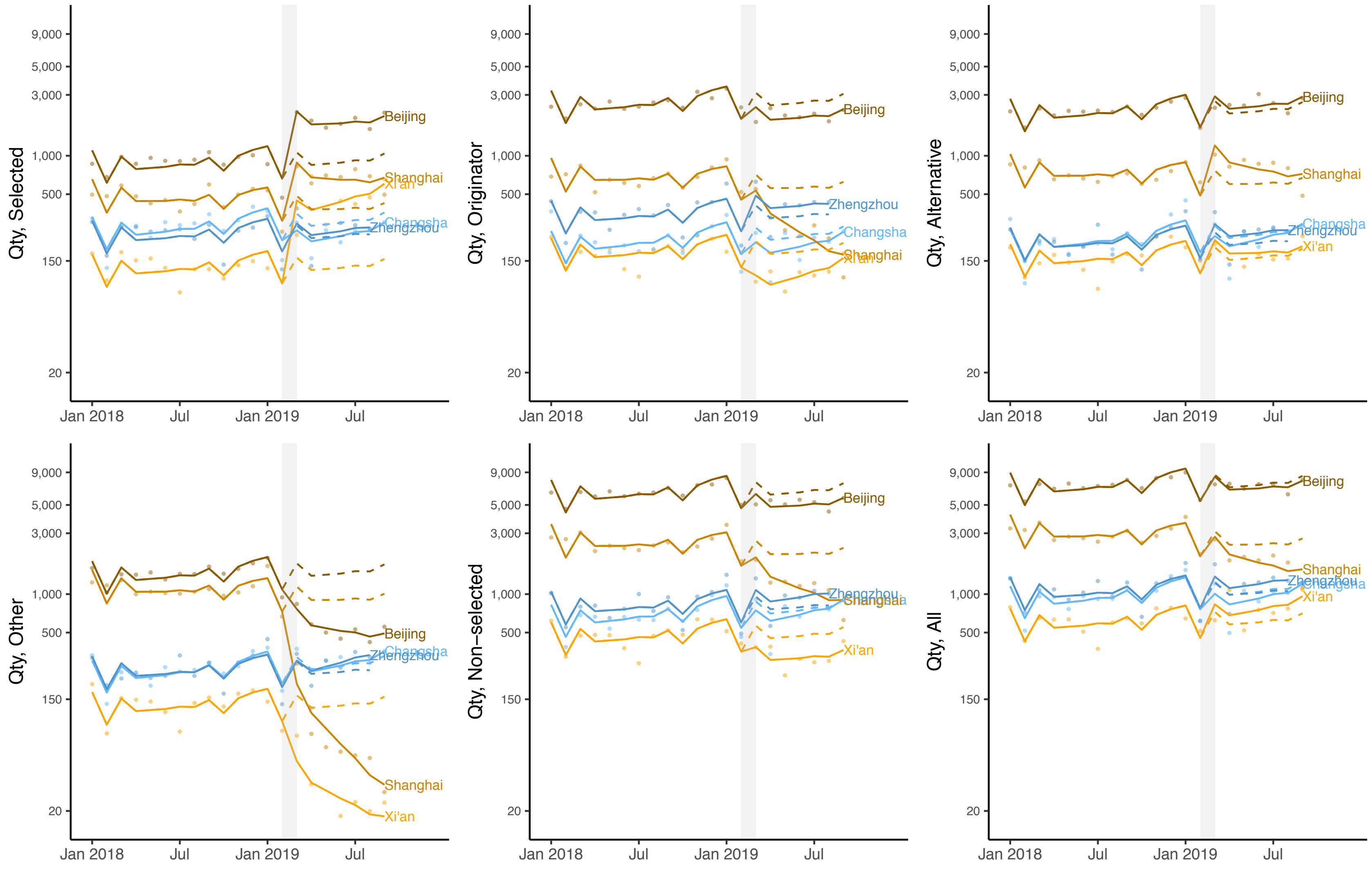

Figure S1. Quantity of drug purchase stratified by cities and drug categories over time. Dots indicate observed monthly purchase quantity (Qty, measured in DDD) per 1, 000 population. The solid lines show the model fitted regression line, and the dashed lines represent model-estimated expected (i.e., counterfactual) purchase had the pilot program not occurred. The observed nadir in drug purchases in a calendar year coincides with the annual lunar new year in February.

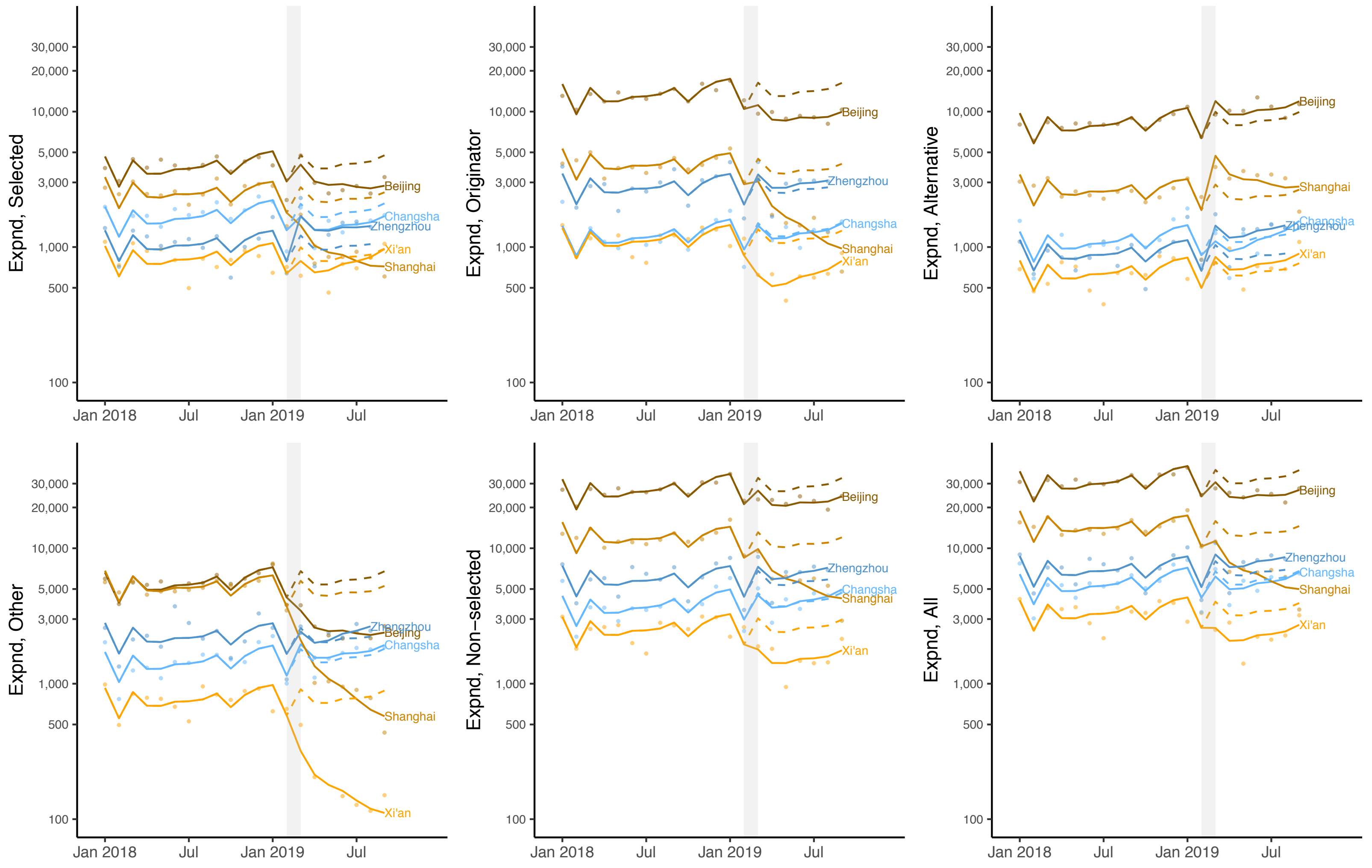

Figure S2. Spending on drug purchase stratified by cities and drug categories over time. Dots indicate observed monthly spending (Expnd, measured in CNY) on drug purchase per 1, 000 population. The solid lines show the model fitted regression line, and the dashed lines represent model-estimated expected (i.e., counterfactual) purchase had the pilot program not occurred. The observed nadir in drug purchases in a calendar year coincides with the annual lunar new year in February.

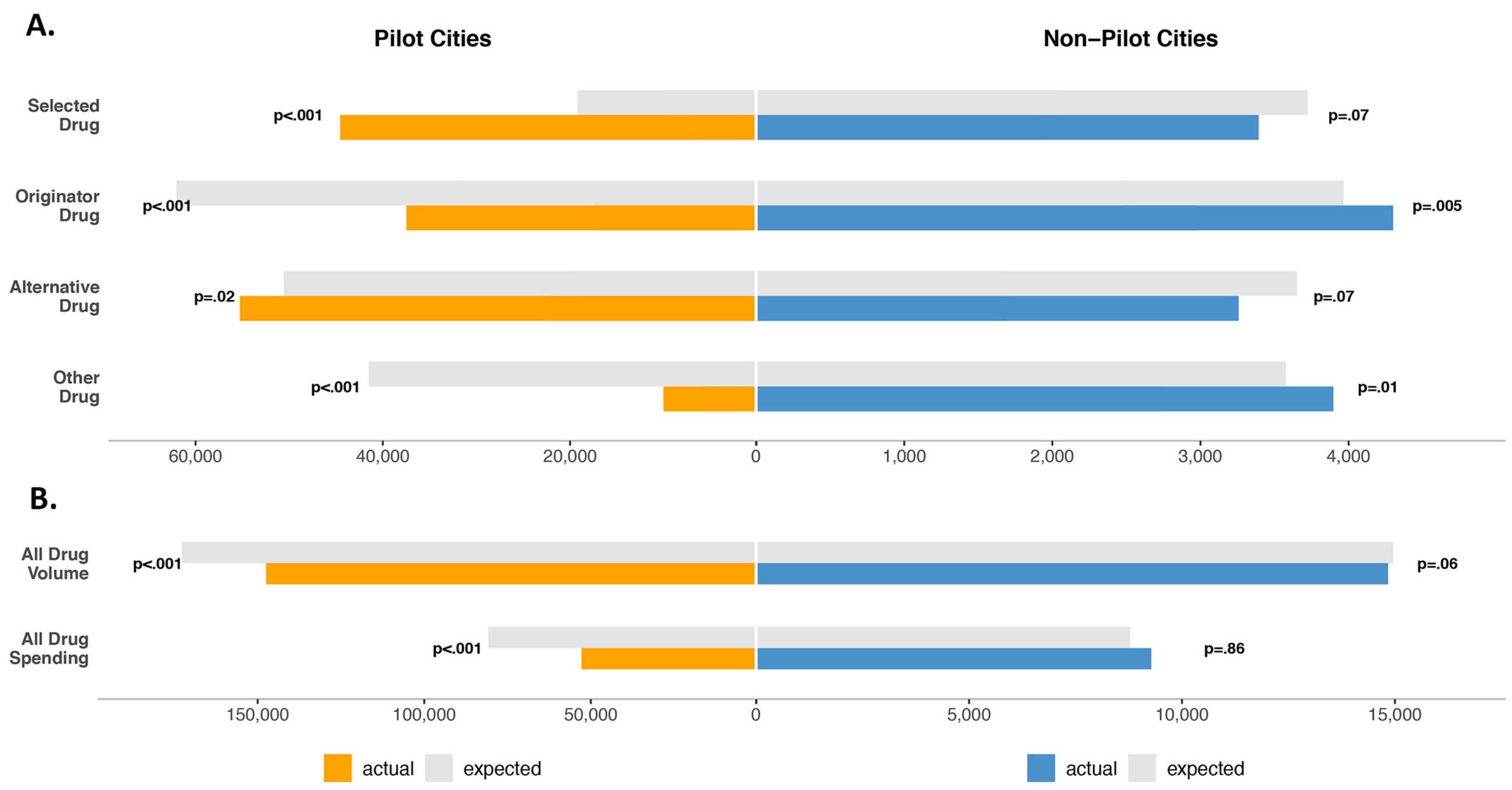

Figure S3. Expected (counterfactual estimate) versus actual (factual estimate) purchase by drug categories, March-September 2019. (A) Expected versus actual purchase quantity (in thousand) by drug categories; (B) Expected versus actual purchase quantity (in 1,000 DDD) and spending (in 100,000 CNY) for all drugs. Specific point estimates for absolute change and the corresponding 95% confidence intervals are provided in Table 2.

Table S2. Observed average monthly drug purchase before and after the pilot program

	Purchase quantity[1] Mean (SD)				Spending on drug purchase[1] Mean (SD)			
	Pre-pilot	Post-pilot	Absolute change	Relative change, %	Pre-pilot	Post-pilot	Absolute change	Relative change, %
**Drug type**								
**Pilot cities[2]**								
All	23863 (3185)	21061 (1492)	-2802	-11·7	10845 (1405)	7541 (818)	-3305	-30·4
Selected	3263 (536)	6358 (529)	3095	94·9	1594 (257)	984 (252)	-609	-38·2
Originator	7529 (1015)	5351 (417)	-2178	-28·9	4069 (501)	2503 (261)	-1566	-38·5
Alternative	6893 (856)	7897 (626)	1004	14·6	2517 (302)	3196 (266)	678	26·9
Other	6177 (1051)	1455 (539)	-4723	-76·5	2666 (446)	858 (287)	-1808	-67·8
**Non-pilot cities[2]**								
All	1956 (539)	1982 (567)	26	1·3	1206 (308)	1236 (340)	30	2·5
Selected	493 (118)	457 (126)	-36	-7·3	256 (62)	261 (79)	5	2·0
Originator	556 (167)	565 (192)	9	1·6	399 (113)	385 (122)	-14	-3·5
Alternative	421 (136)	437 (129)	16	3·8	190 (57)	222 (63)	32	16·8
Other	486 (133)	523 (127)	37	7·6	361 (99)	368 (84)	7	1·9
**All drugs by cities**								
Beijing	15417 (2131)	15287 (1217)	-130	-0·8	6693 (908)	5490 (465)	-1203	-18·0
Shanghai	7822 (1303)	4966 (1204)	-2855	-36·5	3809 (569)	1809 (596)	-1999	-52·5
Xi'an	624 (127)	808 (219)	183	29·3	344 (60)	241 (58)	-103	-29·9
Changsha	835 (238)	829 (205)	-6	-0·7	469 (126)	481 (91)	12	2·6
Zhengzhou	1121 (316)	1153 (476)	32	2·9	737 (189)	755 (300)	19	2·6

[1] Quantity is measured in 1,000 DDD, and spending is presented in 100, 000 CNY

[2] Pilot cities: Beijing, Shanghai and Xi'an. Non-pilot cities: Changsha and Zhengzhou

Table S3 . Interrupted time-series regression model estimates (stratified by drug categories and cities)

	Immediate Change[2]		Monthly Change[2]	
	RR (95% CI)	p-value	RR (95% CI)	p-value
**Selected Drug[1]**				
Beijing	2·55 (2·29, 2·82)	<·001	0·98 (0·97, 0·99)	·006
Shanghai	2·08 (1·69, 2·56)	<·001	0·97 (0·92, 1·03)	·372
Xi'an	2·26 (1·86, 2·74)	<·001	1·17 (1·13, 1·22)	<·001
Changsha	0·75 (0·66, 0·84)	<·001	1·00 (0·98, 1·03)	·992
Zhengzhou	1·24 (0·93, 1·65)	0·144	0·89 (0·80, 0·99)	·026
**Originator[1]**				
Beijing	0·64 (0·60, 0·69)	<·001	1·00 (0·98, 1·01)	·505
Shanghai	0·72 (0·61, 0·85)	<·001	0·82 (0·79, 0·86)	<·001
Xi'an	0·60 (0·50, 0·72)	<·001	1·07 (1·05, 1·09)	<·001
Changsha	0·93 (0·80, 1·08)	·328	1·06 (1·03, 1·09)	<·001
Zhengzhou	1·42 (1·06, 1·89)	·018	0·84 (0·76, 0·93)	<·001
**Alternative Drug[1]**				
Beijing	1·00 (0·93, 1·08)	·967	1·00 (0·98, 1·02)	·919
Shanghai	1·53 (1·30, 1·79)	<·001	0·94 (0·90, 0·99)	·028
Xi'an	1·11 (0·99, 1·24)	·063	1·00 (0·98, 1·02)	·993
Changsha	0·64 (0·53, 0·78)	<·001	1·05 (1·01, 1·09)	·022
Zhengzhou	1·33 (1·01, 1·75)	·041	0·85 (0·77, 0·94)	·001
**Other Drug[1]**				
Beijing	0·45 (0·39, 0·52)	<·001	0·92 (0·90, 0·95)	<·001
Shanghai	0·16 (0·12, 0·22)	<·001	0·76 (0·72, 0·81)	<·001
Xi'an	0·29 (0·20, 0·44)	<·001	0·89 (0·81, 0·99)	·024
Changsha	0·91 (0·76, 1·08)	·277	1·02 (0·98, 1·06)	·387
Zhengzhou	1·50 (1·18, 1·90)	<·001	0·87 (0·81, 0·94)	<·001
**All Non-selected Drug[1]**				
Beijing	0·73 (0·69, 0·77)	<·001	0·99 (0·98, 1·00)	·023
Shanghai	0·73 (0·62, 0·85)	<·001	0·89 (0·86, 0·93)	<·001
Xi'an	0·71 (0·61, 0·83)	<·001	1·01 (0·99, 1·03)	·323
Changsha	0·82 (0·70, 0·98)	·024	1·04 (1·00, 1·08)	·034
Zhengzhou	1·42 (1·09, 1·84)	·009	0·85 (0·78, 0·93)	<·001
**All Drug[1]**				
Beijing	0·91 (0·86, 0·95)	<·001	0·99 (0·98, 0·99)	<·001
Shanghai	0·90 (0·77, 1·07)	·236	0·92 (0·88, 0·96)	<·001
Xi'an	1·11 (0·96, 1·29)	·168	1·07 (1·05, 1·09)	<·001
Changsha	0·80 (0·69, 0·94)	·005	1·03 (0·99, 1·06)	·097
Zhengzhou	1·37 (1·05, 1·78)	·020	0·86 (0·78, 0·95)	·002
**All drug (Spending)**				
Beijing	0·75 (0·72, 0·79)	<·001	0·98 (0·97, 0·98)	<·001
Shanghai	0·73 (0·73, 0·85)	<·001	0·88 (0·84, 0·92)	<·001
Xi'an	0·66 (0·54, 0·81)	<·001	1·05 (1·02, 1·08)	<·001
Changsha	0·97 (0·87, 1·08)	·569	1·00 (0·98, 1·02)	·962
Zhengzhou	1·43 (1·11, 1·83)	·005	0·86 (0·79, 0·94)	·001

[1] Monthly quantity of purchase.

[2] Immediate change refers to the change in March 2019; monthly change refers to the gradual change from April-September 2019.

Table S4. Overall Changes in purchase stratified by drug and cities, March- September 2019

City	Drug	Expected	Model-fitted actual	Absolute Change			Relative Change (%)			p-value
				Estimate	95% CI		Estimate	95% CI		
Beijing										
	Selected	1186	2858	1672	1553	1784	141·5	120·3	163·5	<·0001
	Originator	4951	3133	-1818	-2019	-1619	-36·7	-39·4	-33·8	<·0001
	Alternative	3849	3864	15	-240	262	0·5	-5·9	7·2	0·9000
	Other	2311	830	-1481	-1662	-1313	-64·1	-67·0	-61·0	<·0001
	Non-selected	11103	7835	-3268	-3818	-2731	-29·4	-32·9	-25·8	<·0001
	All	12316	10697	-1619	-2268	-994	-13·1	-17·5	-8·5	<·0001
	All (spending)	54606	38422	-16184	-18643	-13774	-29·6	-32·7	-26·4	<·0001
Shanghai										
	Selected	664	1270	606	453	749	92·9	58·7	132·2	<·0001
	Originator	1217	527	-690	-889	-515	-56·5	-62·7	-49·5	<·0001
	Alternative	1178	1532	353	208	497	30·3	16·4	45·4	<·0001
	Other	1798	157	-1641	-2054	-1287	-91·2	-93·0	-88·9	<·0001
	Non-selected	4159	2219	-1941	-2609	-1347	-46·3	-54·1	-37·6	<·0001
	All	4831	3482	-1349	-2157	-645	-27·5	-38·5	-15·5	<·0001
	All (spending)	24806	12666	-12140	-15609	-9017	-48·7	-55·3	-41·4	<·0001
Xi'an										
	Selected	89	325	236	216	258	267·5	228·8	309·0	<·0001
	Originator	119	87	-32	-51	-14	-26·4	-37·4	-13·7	<·0001
	Alternative	118	131	13	1	24	11·0	0·8	21·9	0·0340
	Other	90	20	-70	-86	-56	-77·7	-82·3	-72·1	<·0001
	Non-selected	324	239	-86	-128	-47	-26·1	-35·2	-16·2	<·0001
	All	413	565	152	98	204	37·1	21·4	54·8	<·0001
	All (spending)	2227	1687	-540	-979	-153	-23·6	-37·1	-8·2	0·0022
Changsha										
	Selected	196	146	-50	-58	-41	-25·3	-28·7	-21·6	<·0001
	Originator	109	120	12	4	20	11·0	3·3	19·4	0·0046
	Alternative	185	138	-47	-61	-33	-25·3	-31·5	-18·8	<·0001
	Other	179	171	-8	-20	4	-4·3	-10·5	2·1	0·1864
	Non-selected	461	430	-31	-61	-2	-6·7	-12·7	-0·4	0·0368
	All	658	576	-81	-119	-45	-12·3	-17·4	-7·1	<·0001
	All (spending)	3456	3357	-99	-249	50	-2·8	-7·0	1·5	0·1906
Zhengzhou										
	Selected	190	169	-21	-54	11	-10·4	-25·4	6·8	0·2096
	Originator	312	269	-43	-103	14	-13·1	-28·8	5·1	0·1508
	Alternative	196	165	-32	-67	2	-15·7	-30·2	0·9	0·0620
	Other	190	190	0	-40	37	0·9	-18·2	23·0	0·9734
	Non-selected	696	623	-73	-206	51	-9·9	-25·9	8·4	0·2510
	All	888	793	-95	-261	59	-10·1	-25·7	7·6	0·2388
	All (spending)	5595	5210	-385	-1331	531	-6·5	-21·4	10·4	0·4172

Note: Quantity of purchase is presented in 10, 000 DDD, expenditure on purchase is presented in 100, 000 CNY.

Methodology: Statistical model evaluating heterogeneous effects in subgroups

$$\log\left(E\left(Y_{i\,j\,t}\right)\right) = \beta_0 + \sum_{base=2}^{I*J} \beta_{base} C_i\, D_j \; + \sum_{slope=2}^{I} \beta_{slope} C_i \, + \sum_{level\_change=2}^{I*J} \beta_{level\_change} C_i\, D_j\, X_t + \sum_{slope\_change=2}^{I*J} \beta_{slopc\_hange} C_i\, D_j \;\; (T - T_0) * X_t + \sum_{m=2}^{12} \beta_m\, M$$

where Y_{jt} is the independent outcome variable (either monthly purchase quantity/spending) for drug type (D) *j* in city (*i*) at time *t* , *T* and *T*- T_0 is the time (month) since the start of the study (January 2018) and the time since the implementation of pilot program (T_0: March 2019) respectively. X_t is an indicator variable where pre-intervention is coded as 0 and post-intervention is coded as 1. *M* is the indicator variable for calendar month used to account for seasonal variation.

The RECORD statement – checklist of items, extended from the STROBE statement, that should be reported in observational studies using routinely collected health data.

	Item No.	STROBE items	Location in manuscript where items are reported	RECORD items	Location in manuscript where items are reported
**Title and abstract**					
	1	(a) Indicate the study's design with a commonly used term in the title or the abstract (b) Provide in the abstract an informative and balanced summary of what was done and what was found		RECORD 1.1: The type of data used should be specified in the title or abstract. When possible, the name of the databases used should be included.<br><br>RECORD 1.2: If applicable, the geographic region and timeframe within which the study took place should be reported in the title or abstract.<br><br>RECORD 1.3: If linkage between databases was conducted for the study, this should be clearly stated in the title or abstract.	Line 45. "We obtained monthly prescription drug purchase data for all purchases from public medical institutions in the three large pilot cities (Beijing, Shanghai and Xi'an) and two non-pilot cities (Changsha and Zhengzhou) between January 2018 to September 2019."
**Introduction**					
Background rationale	2	Explain the scientific background and rationale for the investigation being reported			"However, their analysis only compared the absolute change in the period before and after the policy implementation, without considering confounding by secular trends. In addition, most literature focused on the

					implementation process or policy effect in a single city. No studies have evaluated the implementation of the pilot in other large cities. Therefore, a more comprehensive and rigorous evaluation is crucial to evaluating the effectiveness of the pilot.”
Objectives	3	State specific objectives, including any prespecified hypotheses			“Our study aims to evaluate the impact of the pilot on drug procurement using an interrupted time series analysis with controls in the three large pilot cities (i.e., Beijing, Shanghai, and Xi’an) with a total population of about 60 million. We also examine if the patterns of change associated with the reform differed by cities, drugs, and therapeutic categories. Since the majority of individuals seek care in public hospitals in China, and hospitals automatically convert prescriptions to selected generics, our analysis is generalizable to almost the entire population in these pilot cities. ”
**Methods**					
Study Design	4	Present key elements of study design early in the paper			“We examined changes in the pattern of drug procurement associated with the implementation of the pilot using interrupted time-series (ITS) design, a quasi-experimental design for strong causal inference in the evaluation of population-

					based health interventions introduced at a clear point in time”
Setting	5	Describe the setting, locations, and relevant dates, including periods of recruitment, exposure, follow-up, and data collection			“Our investigators at Wuhan University Global Health Institute obtained data on monthly purchase of each of the 25 bid-winning drugs for pooled procurement (selected drugs) between January 2018 to September 2019. The three pilot cities included Beijing, Shanghai and Xi’an in mainland China.”
Participants	6	*(a) Cohort study* - Give the eligibility criteria, and the sources and methods of selection of participants. Describe methods of follow-up<br>*Case-control study* - Give the eligibility criteria, and the sources and methods of case ascertainment and control selection. Give the rationale for the choice of cases and controls<br>*Cross-sectional study* - Give the eligibility criteria, and the sources and methods of selection of participants<br>*(b) Cohort study* - For matched studies, give matching criteria and number of exposed and unexposed<br>*Case-control study* - For matched studies, give matching criteria and the number of controls per case		RECORD 6.1: The methods of study population selection (such as codes or algorithms used to identify subjects) should be listed in detail. If this is not possible, an explanation should be provided.<br>RECORD 6.2: Any validation studies of the codes or algorithms used to select the population should be referenced. If validation was conducted for this study and not published elsewhere, detailed methods and results should be provided.<br>RECORD 6.3: If the study involved linkage of databases, consider use of a flow diagram or other graphical display to demonstrate the data linkage process, including the number of individuals	“The three pilot cities included Beijing, Shanghai and Xi’an in mainland China. These cities were chosen to represent diverse geographical locations (located in the north, east, and central China, respectively). Two comparable non-pilot cities (Changsha and Zhengzhou) were used to control for potential confounding factors that would affect both pilot and non-pilot cities.”

				with linked data at each stage.	
Variables	7	Clearly define all outcomes, exposures, predictors, potential confounders, and effect modifiers. Give diagnostic criteria, if applicable.		RECORD 7.1: A complete list of codes and algorithms used to classify exposures, outcomes, confounders, and effect modifiers should be provided. If these cannot be reported, an explanation should be provided.	"Two outcome variables were measured in this study: monthly purchase quantity and spending. Purchase quantity was measured by Defined Daily Doses (DDDs), which was developed by the WHO Collaborating Center for Drug Statistics Methodology and used in previous studies…. X_t is an indicator variable where pre-intervention period is coded as 0 and post-intervention period is coded as 1. *M* is the indicator variable for calendar month used to account for seasonal variation."
Data sources/ measurement	8	For each variable of interest, give sources of data and details of methods of assessment (measurement).<br><br>Describe comparability of assessment methods if there is more than one group			"Two comparable non-pilot cities (Changsha and Zhengzhou) were used to control for potential confounding factors that would affect both pilot and non-pilot cities"<br><br>"Each subgroup defined by intervention group (pilot cities versus non-pilot cities), intervention city (Beijing, Shanghai, Xi'an, Changsha and Zhengzhou), drug type (selected, originator, alternative and other drugs), and therapeutic category (cardiovascular disease, mental disorder and cancer) was analyzed separately."
Bias	9	Describe any efforts to address potential sources of bias			"*T* and T- T_0 is the time (month) since the start of the study (January 2018) and the time since the implementation of pilot program (T_0: March 2019)

					respectively. X_t is an indicator variable where pre-intervention period is coded as 0 and post-intervention period is coded as 1. *M* is the indicator variable for calendar month used to account for seasonal variation."
Study size	10	Explain how the study size was arrived at			
Quantitative variables	11	Explain how quantitative variables were handled in the analyses. If applicable, describe which groupings were chosen, and why			"Each subgroup defined by intervention group (pilot cities versus non-pilot cities), intervention city (Beijing, Shanghai, Xi'an, Changsha and Zhengzhou), drug type (selected, originator, alternative and other drugs), and therapeutic category (cardiovascular disease, mental disorder and cancer) was analyzed separately."<br><br>"The three disease categories were chosen because they account for 17 out of the 25 pooled procurement drugs and are the leading causes of disability-adjusted life year in China"
Statistical methods	12	(a) Describe all statistical methods, including those used to control for confounding<br>(b) Describe any methods used to examine subgroups and interactions<br>(c) Explain how missing data were addressed			"Given a disperse variation structure in procurement quantity and skewness in spending, we used negative binomial regression and log-linked Gamma Generalized Linear Model (GLM) for purchase quantity and spending respectively."

		(d) *Cohort study* - If applicable, explain how loss to follow-up was addressed<br>*Case-control study* - If applicable, explain how matching of cases and controls was addressed<br>*Cross-sectional study* - If applicable, describe analytical methods taking account of sampling strategy<br>(e) Describe any sensitivity analyses			"To evaluate heterogeneous effects in subgroups, we examined how the main policy effect terms interacted with each subgroup (see the methods section in the Supplementary Appendix)."
Data access and cleaning methods		..		RECORD 12.1: Authors should describe the extent to which the investigators had access to the database population used to create the study population.<br>RECORD 12.2: Authors should provide information on the data cleaning methods used in the study.	"Yilin Chen, Xu Ji and Hong Xiao had full access to, verified all the data in the study, and take responsibility for the accuracy of the data analysis."<br>"Our investigators at Wuhan University Global Health Institute obtained data on monthly purchase of each of the 25 bid-winning drugs for pooled procurement (selected drugs)"<br>"Analyses were conducted in R-version-4.0.2 (R-Project for Statistical Computing) using data obtained on October 23, 2020."
Linkage		..		RECORD 12.3: State whether the study included person-level, institutional-level, or other data linkage across two or more databases. The methods of linkage and methods of	

				linkage quality evaluation should be provided.	
**Results**					
Participants	13	(a) Report the numbers of individuals at each stage of the study (*e.g.*, numbers potentially eligible, examined for eligibility, confirmed eligible, included in the study, completing follow-up, and analysed)<br><br>(b) Give reasons for non-participation at each stage.<br><br>(c) Consider use of a flow diagram		RECORD 13.1: Describe in detail the selection of the persons included in the study (*i.e.,* study population selection) including filtering based on data quality, data availability and linkage. The selection of included persons can be described in the text and/or by means of the study flow diagram.	"In total, 20.5 billion CNY was spent on 481.5 billion drugs in pilot cities, and 2.6 billion CNY was spent on 41.3 million drugs in non-pilot cities from January 2018 to September 2019 (Table S2)."
Descriptive data	14	(a) Give characteristics of study participants (*e.g.*, demographic, clinical, social) and information on exposures and potential confounders<br><br>(b) Indicate the number of participants with missing data for each variable of interest<br><br>(c) *Cohort study* - summarise follow-up time (*e.g.*, average and total amount)			
Outcome data	15	*Cohort study* - Report numbers of outcome events or summary measures over time<br><br>*Case-control study* - Report numbers in each exposure category, or summary measures of exposure<br><br>*Cross-sectional study* - Report numbers of outcome events or summary measures			
Main results	16	(a) Give unadjusted estimates and, if applicable, confounder-adjusted			The results section

		estimates and their precision (e.g., 95% confidence interval). Make clear which confounders were adjusted for and why they were included<br><br>(b) Report category boundaries when continuous variables were categorized<br><br>(c) If relevant, consider translating estimates of relative risk into absolute risk for a meaningful time period			
Other analyses	17	Report other analyses done—e.g., analyses of subgroups and interactions, and sensitivity analyses			The last paragraph of the results sections.
**Discussion**					
Key results	18	Summarise key results with reference to study objectives			“Over the first seven months of implementation, we found that the VBP pilot program resulted in an increase in the purchase of accredited generics in place of lower quality generics and off-patent branded drugs, resulting in lower total drug purchasing costs.”
Limitations	19	Discuss limitations of the study, taking into account sources of potential bias or imprecision. Discuss both direction and magnitude of any potential bias		RECORD 19.1: Discuss the implications of using data that were not created or collected to answer the specific research question(s). Include discussion of misclassification bias, unmeasured confounding, missing data, and changing eligibility over time, as they	“There are several limitations to this study. First, because the dataset spans a relatively short post-intervention period, our findings only reflected the impact of the VBP policy in the pilot phase and may not generalize to further reform and all settings. More rounds of VBP have been rapidly carried out in the country and assessing long-term trend in purchasing quantity and spending is critical. Although early-stage

				pertain to the study being reported.	evaluation could guide policy makers, healthcare providers and patients to better understand the reform and adapt accordingly, assessing further rounds of large-scale VBP programs after the pilot is equally important. Additionally, the fourteen-month pre-pilot data may not be sufficient to model the pre-pilot or counterfactual post-pilot trends. However, the consistently observed trends and seasonal patterns in the purchase of drugs of any category in all pilot and non-pilot cities, and, to our knowledge, the lack of uncontrolled potential time-varying confounders, suggest that our assumption about the counterfactual post-pilot based on existing information is valid. Lastly, our analysis only focused on drug purchase quantity and health facilities' drug spending. Further research assessing the impact of the VBP policy on other outcomes, including drug prescription/utilization patterns, quality of drug, patients' health outcomes and payments on drugs, as well as the landscape of pharmaceutical industry in China is needed. ”
Interpretation	20	Give a cautious overall interpretation of results considering objectives, limitations, multiplicity of analyses, results from similar studies, and other relevant evidence			China's new VBP policy has demonstrated initial success in containing drug prices and reshaping the composition of the Chinese pharmaceutical market

					in favor of generics of high quality and low prices.
Generalisability	21	Discuss the generalisability (external validity) of the study results			"Since the majority of individuals seek care in public hospitals in China, and hospitals automatically convert prescriptions to selected generics, our analysis is generalizable to almost the entire population in these pilot cities. "
**Other Information**					
Funding	22	Give the source of funding and the role of the funders for the present study and, if applicable, for the original study on which the present article is based			
Accessibility of protocol, raw data, and programming code		..		RECORD 22.1: Authors should provide information on how to access any supplemental information such as the study protocol, raw data, or programming code.	

*Reference: Benchimol EI, Smeeth L, Guttmann A, Harron K, Moher D, Petersen I, Sørensen HT, von Elm E, Langan SM, the RECORD Working Committee. The REporting of studies Conducted using Observational Routinely-collected health Data (RECORD) Statement. *PLoS Medicine* 2015; in press.

*Checklist is protected under Creative Commons Attribution (CC BY) license.
